# Comparative Study on the Outcome of Periorbital Wrinkles Treated with Laser-Assisted Delivery of Vitamin C or Vitamin C Plus Growth Factors: A Randomized, Double-blind, Clinical Trial

**DOI:** 10.1007/s00266-020-02035-z

**Published:** 2020-12-16

**Authors:** Barbara Helena Barcaro Machado, James Frame, Jufen Zhang, Mohammad Najlah

**Affiliations:** 1 Pharmaceutical Research Group, School of Allied Health, Faculty of Health, Education, Medicine and Social Care, Anglia Ruskin University, Bishops Hall Lane, Chelmsford, CM1 1SQ UK; 2 The School of Medicine, Faculty of Health, Education, Medicine and Social Care, Chelmsford Campus, Bishop Hall Lane, Chelmsford, CM1 1SQ UK

**Keywords:** Laser-assisted medication, Laser-assisted drug delivery, Wrinkles, Growth factors, Vitamin C

## Abstract

**Background:**

Despite promising results, laser-assisted drug delivery (LADD) is not yet considered as standard therapies and published data rely mainly on laboratory tests, animal experiments or cadaver skin.

**Objectives:**

This double-blind, prospective, randomized clinical trial investigates the impact in topical application of vitamin C and a cosmeceutical containing growth factors (GFs) on periorbital wrinkles primarily treated with laser skin resurfacing.

**Material and Methods:**

In total, 149 female patients with periorbital wrinkles were consented and randomized into two study groups, R-C (receiving vitamin C only) and R-CGF (receiving vitamin C and a cosmeceutical containing growth factors). The statistical analysis evaluated the efficacy of each treatment regimen using software readouts provided by a three-dimensional stereophotogrammetry system prior to treatment and three months after the procedure. Results were compared to confirm if there was a significant change in the skin roughness and the average depth of the wrinkles between the two groups after treatment.

**Results:**

There was a significant reduction in both skin roughness and average depth of the wrinkles in the group treated with vitamin C and growth factors (*p* <0.01) than those treated with LADD followed by topical application of vitamin C alone. There were no cutaneous reactions or adverse systemic reactions observed in this study related to LADD with vitamin C and GFs.

**Conclusion:**

Controlled laser application might have a great potential to facilitate the absorption of exogenous macromolecules by the skin. Periorbital wrinkles were reduced in both groups, but LADD using vitamin C and GFs provided significantly better results.

**Level of Evidence II:**

This journal requires that authors assign a level of evidence to each article. For a full description of these Evidence-Based Medicine ratings, please refer to the Table of Contents or the online Instructions to Authors www.springer.com/00266.

## Introduction

The senescence of human skin is characterized by the action of physical and biochemical ageing processes within a bi-layer comprised of a rigid superficial stratum corneum (SC), lying on a deeper and less rigid dermis. This phenomenon causes a comparative shrinkage of the more rigid layer over the softer layer, and this leads to the classical appearance of wrinkles. These skin depressions more frequently occur in sun-exposed regions or areas that are subject to repeated movements such as face, neck and hands [1, 2]^.^

Laser skin resurfacing (LSR) delivers a predictable, non-ionizing photoablative radiation that is transformed to thermal energy on the skin surface to improve its tone, texture, wrinkles and pigmentation [3, 4]. Apart from reproducibility and precision, laser has the advantage over other surface ablative measures of conforming to irregular skin surfaces, whether convex or concave.

Several studies demonstrate that lasers disrupt the SC outside-in barrier, disrupt the TJs inside-out barrier and facilitate the bioavailability of molecules through the resulting micro-channels [5, 8]. Most studies, however, rely on in-vitro and ex vivo experiments [9]. In 2018, Badawi and Osman [6] published one of the few reports involving human subjects that confirmed the efficacy of laser-assisted medication. They investigated a laser-assisted transcutaneous delivery of hydroquinone in the hemiface of thirty female patients with bilateral melasma. The other hemiface received hydroquinone only as control. The hemiface treated with LADD exhibited a significant decrease in the degree of pigmentation (*p* < 0.005) compared to the side treated with the hydroquinone only.

The influence of fractional ablative lasers (AFLXs) on skin permeability accrues from some theoretical mechanisms related to Fick's first law of molecular diffusion [7, 10]. This law states that the flux of molecules across a membrane (e.g., the stratum corneum) is a product of the number of molecules available for diffusion, the surface area of the membrane and its thickness. AFLXs influence Fick's first law of diffusion in several ways. Firstly, these devices reduce the thickness of the skin by removing the SC and thus decrease the diffusion path length [10–12]. Secondly, fractional ablative lasers increase the diffusion area by producing microthermal zones and allow the permeant to spread into deeper strata and penetrate laterally toward the residual thermal damage zone [10]. The damaged SC permits the penetration of molecules of high molecular weight into deeper layers and hydrophilic drugs to succeed in diffusing through the lipid-rich layer [3].

As the periorbital area is not flat, the laser beam collimation provides regular skin penetration. Although devices such as microneedling may be less expensive as skin physical penetration enhancers, variables such as the different assembly of needles composing the devices available in the market and the operator-dependent employment of strength during microneedling application may cause different depths of penetration. Finally, the high local temperature generated by the laser generates accentuated molecular motion and consequent cutaneous permeation of any topically applied medication toward deeper layers [13–17]. This tissular heat explains the increased efficiency in drug absorption using AFXLs over other physical penetration enhancers [16, 17].

If there is an insufficient accumulation of a medical substance in the stratum corneum, the permeant diffuses into deeper strata by concentration gradient [13]. As the drug enters the laser channels and penetrates toward the dermis [12, 13, 18], there may be initial concerns about systemic absorption and later risk of bacterial infection caused by the direct exposure of underlying dermis and its vasculature to the outside environment [17].

The immune system within skin involves a well-coordinated cell-mediated and humoral immune response to potentially harmful agents, including drugs [5, 9, 12]. This immune response can result in cutaneous intolerance, hypersensitivity reactions and contact dermatitis, which can further provoke failure of transcutaneous medication [19].

To elucidate these concerns, clinical studies in humans are paramount because the currently available ex-vivo studies and experimental animal models are unsuitable [19–21].

The aim of this double-blind, prospective, randomized clinical trial was to investigate the impact from the topical application of different substances on skin surface immediately after laser skin resurfacing and to determine if adding growth factors to the skin surface would prove beneficial. With this goal, two study groups were analyzed, one control group received vitamin C, and the study group received growth factors and vitamin C. These substances were applied onto laser resurfaced skin wrinkles, immediately after the procedure and kept under occlusion for 30 min.

The primary endpoint was the obtention of readouts related to skin roughness (Rgh) and average depth (AD) of facial skin wrinkles provided by a three-dimensional stereophotogrammetry system (LifeViz^TM^ Micro, Quantificare, France). This system is linked to the software Dermapix^®^ which quantified the change in the wrinkle's microtopography between baseline and 3 months after LADD. The efficacy of each treatment regimen was statistically analyzed and compared to assess significant differences in the skin roughness and the average depth of the wrinkles between the two groups.

## Material and Methods

The sample size calculation for this study was based on a pilot study performed in 2017 as part of the PhD studies of the first author. That study estimated the *n* of 44 patients for each study group to have 80% power to detect the mean difference of 0.1149 between the two related samples (SD = 0.2635). A two-sided paired *t*-test was used with a significant level of 5%. In total, 149 female patients with Fitzpatrick skin types I–IV, aged between 43 and 70 years, seeking laser treatment for periorbital wrinkles were recruited, consented and randomized into two study groups, R-C (receiving vitamin C only) and R-CGF (receiving vitamin C and a cosmeceutical containing growth factors).

The exposure to the chemical agents in each group was limited to one session and only to the facial region. The vitamin C (Vitasantisa^®^) is approved for intravenous use and licensed by Health Ministry, Brazil. Vitamin C has demonstrated a beneficial effect on skin ageing [2, 22, 23], and no toxicity has been reported when this water-soluble vitamin was applied as part of LADD [24].

Growth factors were included in this LADD study because there was evidence in the literature to support their effectiveness after intravenous application as early as 1999 [26]. GFs have been rarely investigated as adjuncts to skin rejuvenation. In theory, the artificial supplementation of human GFs in vivo can promote skin rejuvenation [1, 26–29] and no report of allergic reactions related to their topical use was found in the literature. The cosmeceutical TNS Recovery Complex^®^ (SkinMedica, Carlsbad, CA, USA) used in group R-CGF contained a mixture of GFs and cytokines (VEGF, PDGF, HGF, IL-6, IL-8, and TGF-β1) as active ingredients. The cosmeceutical was chosen according to the Faculty Research Ethics Panel's request for a product approved by the CE (European Conformity) and commercialized for topical use [25]. As the cosmeceutical has a patented composition, we were not able to describe the concentration of each growth factor isolated.

Patients whose periorbital area had been treated with botulinum toxin for up to 4 months prior to the study, or to laser treatment, dermabrasion or chemical peeling up to 6 months before the study were excluded.

### Study Design

The enrolled patients signed a consent form on the day of treatment and were randomized into either group R-C or R-CGF. A 3D stereophotogrammetry digital camera (LifeViz^TM^ Micro, Quantificare, France) was used to photograph the relevant anatomical areas before and 3 months after the treatment. A laser tape measure was employed to standardize the position of pre- and post-operative images by using a vertical line extending from the lateral eyebrow tail toward the jawline and horizontally from the eyebrow tail toward the temporal hairline.

After photographic documentation, an anesthetic ointment composed of lidocaine 7% and tetracaine 7% was applied to the targeted areas for 30 min. Immediately before the procedure, 20 mg of oral prednisolone and 10 mg of ketorolac tromethamine were given to all patients. The eyes were protected with moist gauze and goggles.

All patients were submitted to a single session of a 2.940 nm Erbium-Yag fractional ablative laser treatment by the first author (Starlux^®^ 500 Palomar Inc., Burlington, MA).

The same laser protocol was applied to all patients (Table 1). The short pulse targeted cutaneous ablation, and the long pulse intended tissue coagulation. The blue optics used in this study scans with a spot size of 6 x 6 mm^2^ and produces densities of 169 vertical microperforations (microbeam size: 100–140 µm) per pass, or yet, 469 microperforations per cm^2^. Considering that 1 cm^2^ has 100,000,000 µm^2^, one laser pass with the blue optics performs a total area of 56,280 µm^2^ of microperforations or a density of approximately 5.6%. The separation between the centers of each microchannel was calculated as 500 µm, and the diameter of the microchannel opening at the skin surface was around 100 µm.

**Table 1:** Fractional Erbium:Yag laser parameters protocol

Handpiece/optics	Energy short pulse (250 ms)^***^	Energy long pulse (5 ms)	Density of microbeams (μb/cm^2^)	No. of passes	Microbeam size
2940 nm, Blue optic 6x6 mm	9 mJ^*^/μb^**^	8 mJ/μb	469	4	120–140 μm

^*^mJ = milijoules (1 mJ = 0.001 Watt-sec)

^**^μb = microbeams (structure of optical spots through which light radiation is emitted)

^***^ms = miliseconds

Immediately after the laser treatment, 200 mg of vitamin C was applied on the skin to patients in group R-C. Patients in group R-CGF underwent topical application of vitamin C plus the cosmeceutical containing GFs. The treated areas of skin being investigated skin were occluded for 30 min and protected from light exposure to avoid the photodegradation of vitamin C. Both the researcher and the patient were unaware of the randomized treatment given (double-blind study).

Patients were discharged and instructed to: (1) clean the treated area with a saline solution once a day, (2) to cover the treated area with dexpanthenol (Bepantol®–Bayer) 4 times a day until the cutaneous debris have entirely disappeared, (3) to take Fexofenadine once a day for 5 days and (4) not to apply cosmeceuticals and other topical medications on the face until the follow-up visit. Valaciclovir prescription was restricted to patients with the previous history of herpes simplex.

Patients were monitored for adverse events. Three months after the treatment (from January 2019 to January 2020), post-procedure photographs of the periorbital area were taken, uploaded to the computer and transferred to the software Dermapix^®^. The pre- and post-procedure images were synchronized for comparison and converted into three-dimensional images. A contour comprising periorbital wrinkles was designed, and the software delivered information on skin roughness (Rgh) and wrinkle's average depth (AD) before and after LADD.

### Statistical Analysis

Data delivered by the software Dermapix^®^ were analyzed by the software package SPSS IBM (Version 26.0 IBM Corp^©^ for Mac, Armonk, New York, USA). Tests were applied to compare and correlate skin roughness (Rgh) and wrinkle average depth (AD) measurements, pre- and post-procedure.

The histograms and statistical tests for normality (Shapiro-Wilk and Kolmogorov-Smirnov) confirmed that the variables had a non-Gaussian distribution in at least one group. Data were summarized by quartiles, median, mean and SD of the numerical variables under study. The inferential analysis involved non-parametric-related samples tests (Wilcoxon Signed Rank Test) and independent sample tests (Mann-Whitney U test). Spearman rho was utilized to establish the correlation between wrinkle average depth (AD) and skin roughness (Rgh) improvement. The criterion for determining significance was set at 5% and the findings were considered significant with a *p*-value < 0.05.

## Results

Skin crusting lasted from 5 to 8 days. Early moderate periorbital swelling was revealed in all patients and spontaneously resolved by the third-day post-procedure. Post-laser erythema appeared after the seventh day after the procedure and subsided by the twentieth day. Hypochromia and herpetic eruptions were not detected.

### Group R-C

**Fig. 1:**
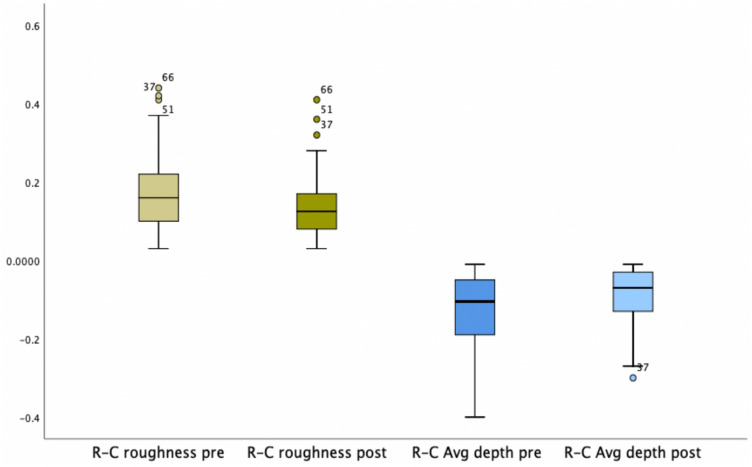
Boxplots representing the reduction in skin roughness and wrinkle average depth in group R-C

Of the 149 patients included in the study and randomized according to the protocol, 74 patients were followed up in group R-C. The age of the patients varied between 43 and 70 years old (mean 57.1 years ± 6.7 SD). The median of AD was 0.1 mm [Interquartile Range (25th–75th percentile) (IQR) 0.19–0.05 mm] pre-procedure, whereas the post-procedure measurements showed a median of 0.07 mm (IQR 0.13 to 0.03 mm). This variation is represented in Fig. 1.

Wilcoxon Signed-Rank Test (Sig. 2-sided test) showed that the reduction of Rgh and the AD were significant with a *p* <0.01 (Table 2). Pre-procedure, the Rgh was 0.16 (IQR 01–0.22) compared to 0.13 (IQR 0.08–0.17) post-procedure. The percentage of parameter modification (∂ reduction) established the variation between the pre- and post-measurements of a given parameter and was calculated by the mathematical formula:

**Table 2:** Descriptive analysis of the variables roughness (Rgh) and average depth (AD) in group R-C (*n =* 74)

Variable	Group R-C (*n =* 74)										
	Pre-treatment					Post-treatment					Wilcoxon signed-rank test
	Mean/SD	Median	IQR (25th–75th percentiles)	Min	Max	Mean/SD	Post median	IQR (25th 75th percentiles)	Min	Max	*p*-value
Age (years)	57.1 ± 6.7	57	52.0–62.3	43	70	57.1 ± 6.7	57	52.0–62.3	43	70	n/a
Rgh	0.17 ± 0.09	0.16	0.10–0.22	0.03	0.44	0.14 ± 0.08	0.13	0.08–0.17	0.03	0.41	< 0.01
AD	− 0.13 ± 0.09	− 0.11	− 0.19 to − 0.05	− 0.4	− 0.01	− 0.09 ± 0.07	− 0.07	− 0.13 to − 0.03	− 0.30	− 0.01	< 0.01
Rgh ∂ reduction (%)						15.5	18.8	7.5–29.1	− 27.3	59.5	n/a
AD ∂ reduction (%)						30.5 ± 30.7	33.3	8.5–50.0	− 66.7	83.3	n/a
Simultaneous computation of AD and Rgh ∂ reduction (%)						49.5 ± 35.7	50.1	18.8–72.3	− 31.3	132	n/a

*IQR* Interquartile Range (25th–75th percentile)

*Min* Minimum; *Max* Maximum; *n/a* not applicable

∂ reduction (%) = (pre - post)/pre × 100

The simultaneous computation of both parameters (Table 2) corresponded to the sum of the ∂ reduction of Rgh and AD. Variables in patients submitted to LADD with vitamin C (group R-C) showed that the Rgh presented a median ∂ reduction of 18.8% (IQR 7.5–29.1%) and the AD underwent a median ∂ reduction of 33.3% (IQR 8.5–50%). The median of the simultaneous computation of Rgh ∂ reduction + AD ∂ reduction was 50.1% (IQR 18.8–72.3%)

**Fig. 2:**
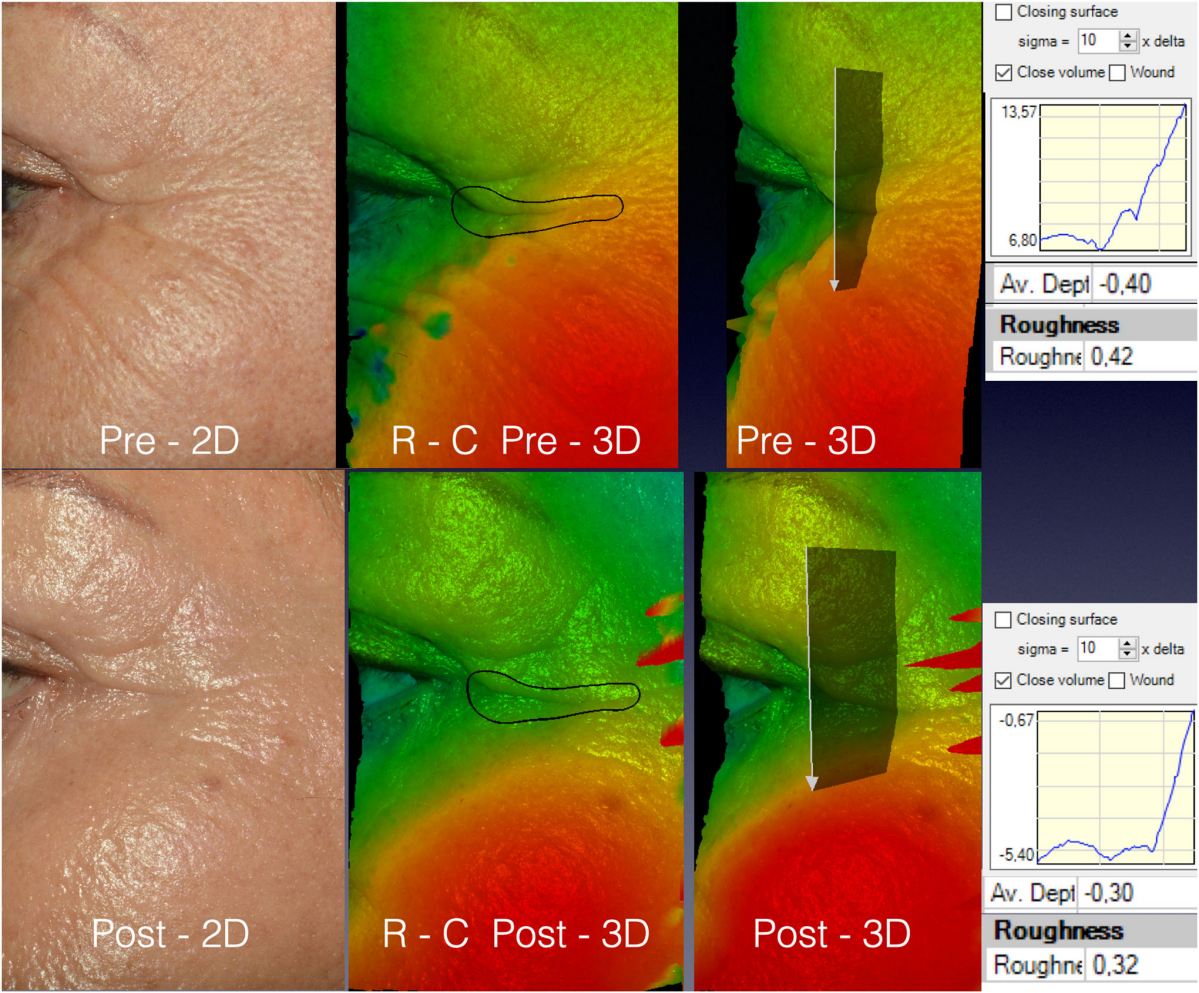
A 69-year-old patient from group R-C. The post-procedure images were obtained the 94th day. The software permitted the rotation of the images, and this provided a different perspective and visualization of the periorbital skin roughness modification. The graphs on the right correspond to the skin roughness under the designed arrow

Figure 2 shows bi- and three-dimensional pictures of a patient from group R-C. The delimited wrinkle exhibited Rgh reduction by 25% (from 0.42 to 0.32) and the AD diminished by 25% (from 0.4 to 0.3 mm). The 3D images on the right were rotated so that graphics on the right could show the variation of skin roughness in the area underlying the white arrow.

### Group R-CGF

From the 149 randomized participants, all 75 patients composing group R-CGF were followed-up at 3 months. Participants were aged from 43 to 70 years old (mean 58.05 ± 6.8 years) (Table 3).

**Table 3:** Descriptive analysis of the variables roughness (Rgh) and average depth (AD) in group R-CGF (*n =* 75)

	Group R-CGF (*n =* 75)										
	Pre-treatment					Post-treatment					Wilcoxon rank test
Variable	Mean/Sd	Median	IQR (25th–75th percentiles)	Min	max	Mean/SD	Median	IQR (25th–75th percentiles)	Min	Max	*p*-value
Age (years)	58.05 ± 6.8	57.0	53.0 to 63.0	43.0	70.0	58.05 ± 6.8	57.0	53.0 to 63.0	43.0	70.0	n/a
Rgh	0.26 ± 0.18	0.22	0.15 to 0.34	0.06	1.31	0.13 ± 0.1	0.11	0.08 to 0.16	0.04	0.61	< 0.01
AD	0.19 ± 0.14	0.15	− 0.29 to − 0.09	− 0.56	− 0.01	− 0.08 ± 0.08	0.06	− 0.11 to − 0.02	− 0.41	− 0.01	< 0.01
Rgh ∂ reduction (%)	n/a	n/a	n/a	n/a	n/a	45.8 ± 17.4	45.0	34.4 to 58.3	4.7	79.0	n/a
AD ∂ reduction (%)	n/a	n/a	n/a	n/a	n/a	57.2 ± 27.3	62.5	34.5 to 81.8	− 2.6	95.8	n/a
Simultaneous computation of AD and Rgh ∂ reduction (%)	n/a	n/a	n/a	n/a	n/a	103 ± 37.4	105.5	74.6 to 134.3	2.1	165	n/a

*IQR* interquartile range (25th–75th percentile)

*Min* Minimum; *Max* Maximum; *n/a* not applicable

**Fig. 3:**
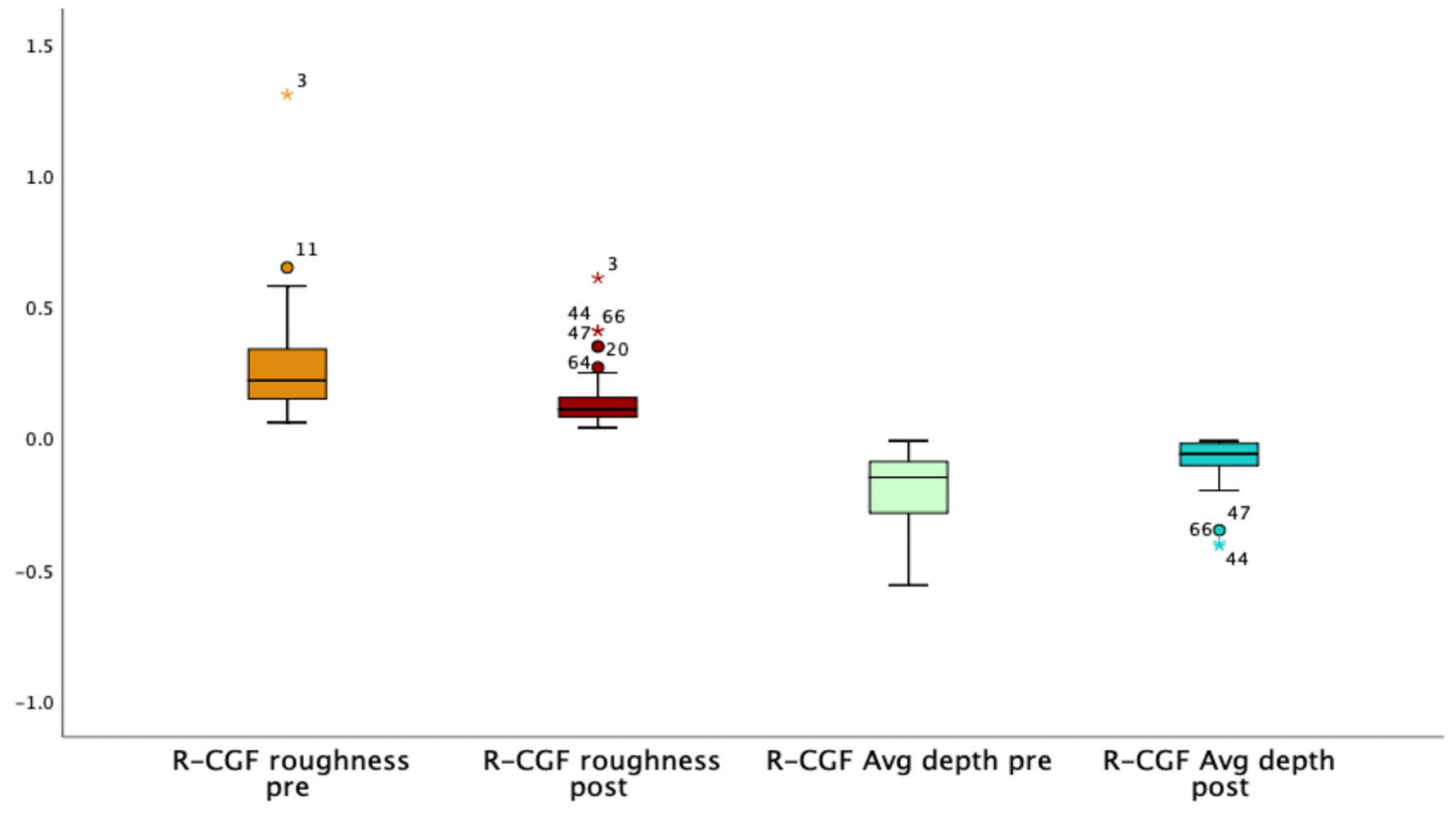
Boxplots representing the modification of skin roughness and wrinkle average depth of group R-CGF

The Wilcoxon signed-rank test confirmed that the mean difference of the pre- and post-procedure data was significant with a *p* <0.01 for both variables. Pre-procedure, the median of Rgh was 0.22 (IQR 0.15–0.34) compared to 0.11 (IQR 0.08–0.16) post-procedure. The median of AD was 0.15 mm (IQR 0.29 to 0.09 mm) pre-procedure, whereas the post-procedure AD_CGF_ showed a median of 0.06 (IQR 0.11– 0.02 mm) (Fig. 3).

Patients treated with LADD and topical application of vitamin C plus the cosmeceutical containing GFs (group R-CGF) presented median of the Rgh_CGF_ ∂ reduction by 45% (IQR 34.4–58.3%) and the median of AD ∂ reduction by 62.5% (IQR 34.5–81.8%). The median of the simultaneous computation of the Rgh ∂ reduction + AD ∂ reduction in was 105.5% (IQR 74.6–134.3%).

**Fig. 4:**
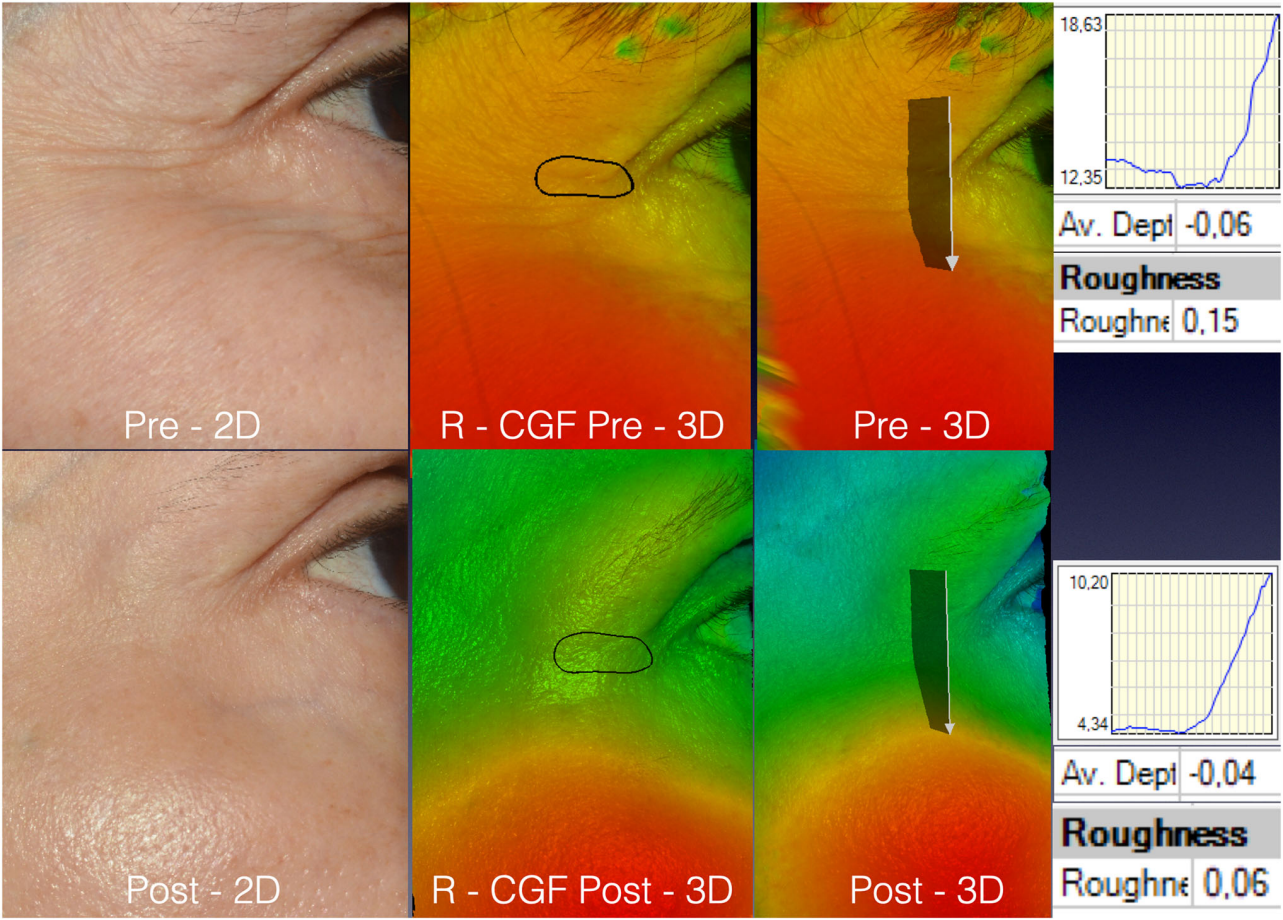
The pictures of a 57-year-old patient from group R-CGF. Pictures were obtained on the 90th day post-procedure. The software permitted the rotation of the images and graphics that illustrated the variation in the skin roughness pre- and post-procedure

Figure 4 exhibits pictures of a patient submitted to LADD with vitamin C and the cosmeceutical containing GFs. The visual skin improvement was confirmed by the data delivered by the 3D SPM system (on the right). Rgh reduced by 60% (from 0.15 to 0.06) and AD diminished by 33.33% (from 0.06 to 0.04mm).

### Comparison between Groups R-C and R-CGF

Spearman's correlation coefficient (Sig 2-tailed) was used to analyze the correlation between both variables. The negative correlation between the Rgh and AD was significant in both groups (*p* < 0.001). In group R-C, rho was 0.808 pre-procedure and became 0.780 post-procedure. The negative correlation also became weaker in group R-CGF, with a rho = 0.770 pre-procedure and rho = 0.680 post-procedure. This correlation represents the tendency to skin flattening and suggests that LADD was able to interfere with both variables at the same time.

According to the Mann-Whitney test (Table 4), the ∂ reduction of AD, Rgh and the simultaneous reduction in both parameters were significantly higher in group R-CGF (*p* <0.01). These findings confirmed that patients submitted to LADD, using vitamin C and GFs blended into a cosmeceutical, produced statistically significant better results than those treated with LADD followed by vitamin C alone.

**Table 4:** Descriptive analysis of the variables (roughness and average depth) according to the two treatment regimens–Mann-Whitney test

Variable	Group C (*n* = 74)		Group CGF (*n* = 75)		*p-*value
	Median	IQI	Median	IQI	
Age (years)	57.1	52.0 to 62.3	58.1	53.0 to 63.0	0.39
Rgh pre-procedure	0.16	0.10 to 0.22	0.22	0.15 to 0.34	<0.01
Rgh post-procedure	0.13	0.08 to 0.17	0.11	0.08 to 0.16	0.50
Rgh ∂ reduction (%)	18.8	7.5 to 29.1	45.0	34.4 to 58.3	< 0.001
AD pre-procedure	− 0.11	− 0.19 to − 0.05	− 0.15	− 0.29 to − 0.09	= 0.001
AD post-procedure	− 0.07	− 0.13 to − 0.03	− 0.06	− 0.11 to − 0.02	0.27
AD ∂ reduction (%)	33.3	8.5 to 50.0	62.5	34.5 to 81.8	< 0.001
Simultaneous computation of AD and Rgh ∂ reduction (%)	50.1	18.8 to 72.3	105.5	74.6 to 134.3	< 0.001

*IQI* interquartile interval (Q1–Q3); Mann-Whitney test

No bias resulted from gender variation or age heterogeneity because all patients were females, and the age variation between both groups was not significant (*p* = 0.39).

**Fig. 5:**
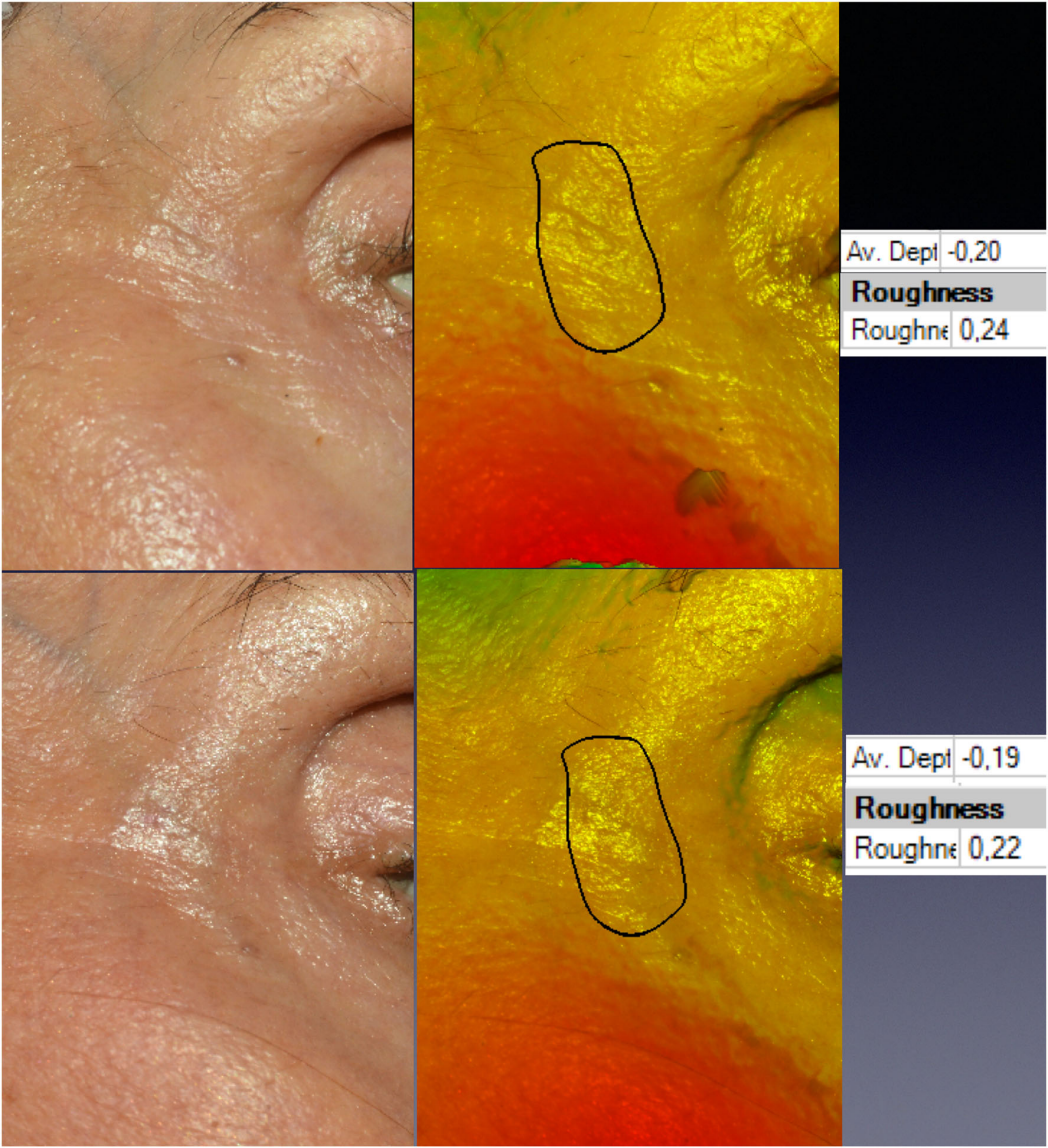
The bi- and three-dimensional pictures of a 68-year-old patient submitted to LADD with vitamin C. Data on the right were collected by the 3D SPM at the 90th day post-procedure and concern the relief of the periorbital vein

**Fig. 6:**
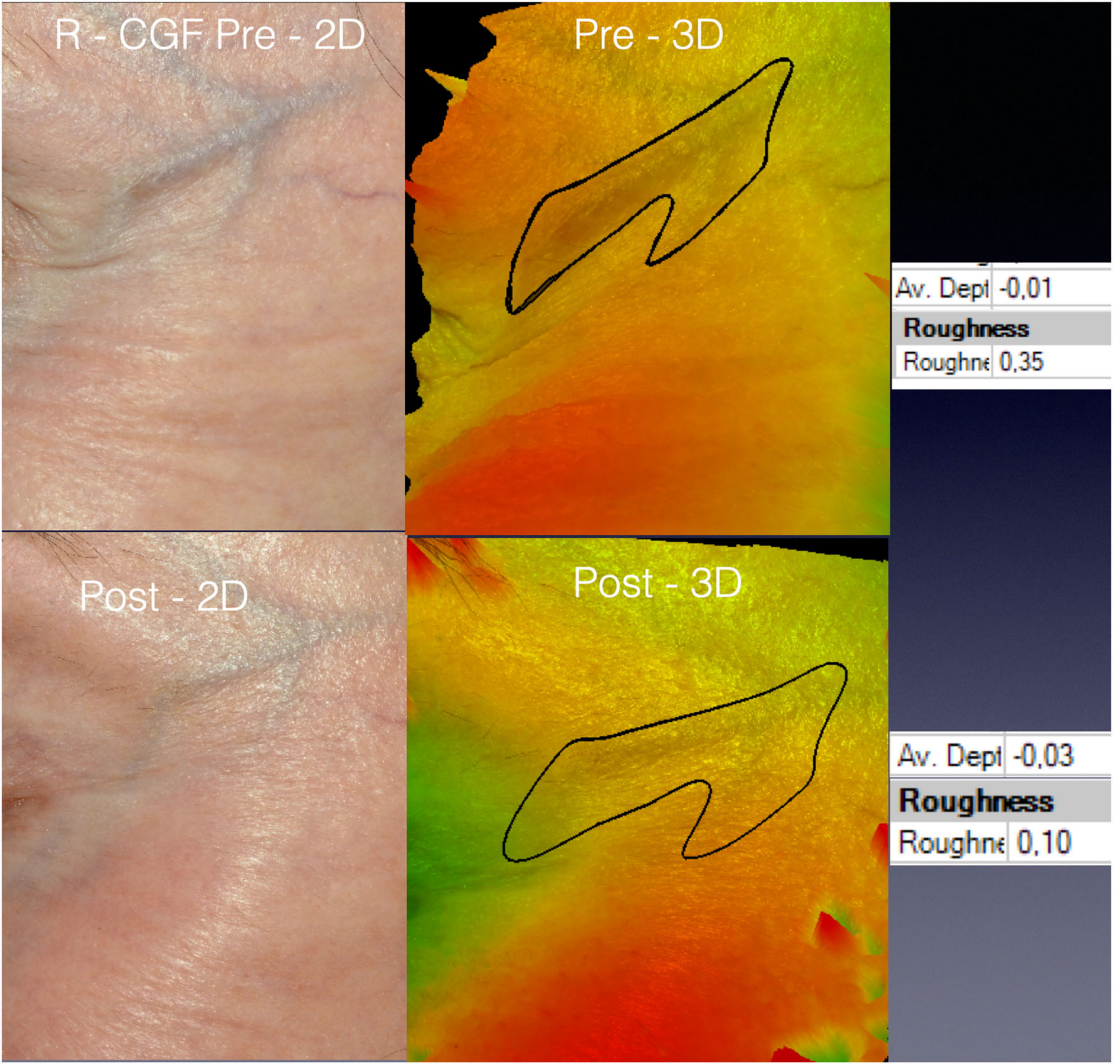
Pictures of a 66-year-old patient submitted to LADD with vitamin C and the blend of GFs. The post-procedure images were obtained on the 93th day. The prominent temporal superficial veins became less visible after the treatment

The concealment of protuberant vessels involving the application of GFs was confirmed as a secondary outcome by the statistical analysis and the clinical examination. Figure 5 shows one patient from group R-C who presented conspicuous periorbital veins. The skin improvement occurred but was not sufficient to conceal those vessels. On the other hand, Fig. 6 presents a patient from group R-CGF in which the roughness of the area comprising the prominent temporal superficial veins reduced from 0.35 to 0.1, which corresponded to Rgh ∂ reduction of 71.4%. The wrinkle selected for analysis showed a simultaneous reduction of AD and Rgh 90%. As the prominent vessels cause elevations in an area that usually presents a regular surface and discrete demotions, the enhancement of the skin thickness has permitted for the correction of the undesired elevation and the return of the skin cover to a slight natural depression. That is the reason for the increased average depth in the post-treatment photograph in Fig. 6.

## Discussion

Facial rejuvenation surgical procedures naturally provide more impressive visual benefits to the patients as they remove skin in the scale of centimeters. However, addressing the skin cover on facial areas has become an important ancillary procedure, especially when the sun exposure, or other extrinsic or intrinsic factors have led the skin to present alterations in its microstructure in the form of mottled pigmentation and wrinkles.

The development of fractional ablative lasers (AFLXs) has permitted for safe and effective skin rejuvenation. AFXLs produce partial ablation of the SC and microperforations into the dermis that can potentially be used as a physical penetration enhancer to treat several skin conditions. Although the use of lasers is not compulsory to improve skin quality and is expensive, when a well-established protocol is used, the precision of the laser computerized system eliminates potential technique-dependent bias regarding the depth of skin microperforation.

The percentage of skin treated with AFXLs and subsequent tissue reaction are dictated by the energy output, density setting, the number of times that the laser hits the target tissue (pulse repetition) and the pulse duration [8, 11, 23]. The density is the number of (microthermal thermal zones) MTZs produced by the laser per unit area (cm^2^), and it varies with the number of laser passes. The lasers settings also influence laser-tissue-drug interaction and consequent drug delivery and bio-distribution [11, 17, 30].

The concentrations of the product in skin aiming at laser-assisted medication or laser-assisted drug delivery are reported to stabilize when densities up to 5% are reached [16, 17, 31]. In 2014, Sklar et al. found that the application of low densities facilitates optimal intracutaneous drug accumulation and that the use of higher densities led to significant reductions in both intra- and transcutaneous delivery per single MTZ [8]. However, that was an ex vivo study, and this type of investigation neglects the dermal dynamic blood flow, which may be responsible for the absence of drug saturation in vivo.

The laser protocol utilized in this study proved efficient in providing drug penetration of macromolecules (GFs and cytokines). We have restricted the number of passes over the same skin surface area to 4 times because the target chromophore (water) reduces after each pass. Several laser passages over the tissue increase the risk of thermal injury and neither enhance the drug uptake nor the effectiveness of the treatment [8, 23, 32].

According to the literature, another factor that can impact the result of the treatment is the time lag before applying the medication, because the spatiotemporal closure of AFXL-induced channels occurs within 24–48 hours after laser exposure. The dermis can quickly become inaccessible owing to the deposition of debris, fibrin, inflammatory mediators and keratinocytes inside the microchannels [16]. Both study groups underwent transcutaneous medication during the first 30 min post-procedure, after gentle skin cleaning. This thirty-minute period was recently confirmed as the optimal interval for LADD [15]. Nonetheless, any residual disruption of cutaneous layers can still be observed 3 weeks after LSR [4, 10, 11]. This time-lapse must be evaluated in future studies to establish the therapeutic window for the topical delivery of medication.

Despite the promising results, previous clinical studies have emphasized the theoretical risk of induced systemic toxicity [11, 33]. This highlights questions linked to regulatory approval and has limited further objective research and restricted the commercialization of active delivery products [20, 34]. It is difficult, if not impossible, to determine cutaneous drug penetration based solely on molecular properties when there are other confounding factors such as dietary intake, endogenous production of substances, variable blood flow and the complex, new surface area and geometry created by the laser-induced microchannels. Therefore, clinical trials are essential to determine the safety of this therapeutic modality. To date, no adverse toxicity has ever actually been linked to LADD [7, 16, 26, 35].

The pharmacological supplementation of GFs is described to exert a therapeutic benefit to scarring and skin senescence because the artificial is supposed to mimic the physiological, molecular biology process to promote skin rejuvenation and enhance the self-healing capacity [1, 26–29]. However, investigation on human subjects are scarce, and some studies involved a low number of patients, which reinforces the importance of this clinical study. In 2006, Erlich et al. published a blinded comparative study on 12 healthy females with facial wrinkles (mean 50 years-old) [26]. The patients elected one hemiface to apply a cosmeceutical containing transforming growth factor beta 1 (TGF-β1), L-ascorbic acid, and *Cimicifuga racemosa* extract and the other side received the cosmeceutical used in the present clinical study. Both products provided significant improvement in facial rhytids, but the authors suggested that the supplementation of L-ascorbic acid (vitamin C) was essential in proportioning skin improvement [26].

After thermal and physical trauma such as laser skin resurfacing, the fibroblasts replace the initial ECM under the influence of GFs, producing type III collagen and adult/mature type I collagen in the scar. Type IV collagen is produced at the dermal-epidermal junction. GFs bind to their specific receptors on the cell surface and this interaction activates several molecular events that are essential for wound healing and tissue repair [1, 24]. The major GF families involved in these processes are the epidermal growth factor (EGF), keratinocyte growth factor (KGF), fibroblast growth factor (FGF), platelet-derived growth factor (PDGF), TGF-βs (transforming growth factor beta), heparin-binding growth factor (HGF) and vascular endothelial growth factor (VEGF). These GF families (i) regulate the growth, differentiation, proliferation and cellular influx of fibroblasts and monocytes, (ii) affect collagen and ECM biosynthesis and (iii) promote neoangiogenesis [1, 4].

GFs poorly penetrate the skin because they are hydrophilic proteins composed of hundreds of amino acids with molecular size larger than 15000 Da [1]. Another restriction to using GFs within any transcutaneous medication is related to their low stability [36, 37]. The effectiveness of GFs is quickly nullified due to clearance from the site by diffusion, or inactivation by proteolytic cleavage. If the degradation is excessive, the biomolecules may not exist at sufficient concentrations to exert their functions. As the mode of delivery of GFs may interfere with the therapeutic success [37], this study confirms that the incorporation of GFs as part of LADD can be efficient and does not require supraphysiologic doses of GFs to ensure a sufficient active concentration. For ethical reasons, it was not possible to use GFs as part of a composition with known concentrations and obliged the use of a blend of GFs contained in a patented formula.

Based on the already established knowledge that the lasers influence drug delivery and consequent bio-distribution [11, 17, 30], the objective of this study was to determine if the addition of growth factors to the skin immediately after laser skin resurfacing would impact the result of the treatment. As the effect of LSR in skin rejuvenation has already been demonstrated, there was no need to investigate the effect of laser alone [3, 11, 22]. Therefore, the group receiving vitamin C only was established as the control group. The group receiving two medications (vitamin C and the cosmeceuticals containing GFs) presented statistically significant better results than the control group, which was medicated with vitamin C only. Although it is not possible to establish to what extent the by-pass of the epidermal layers protects the GFs from degradation, this is an important finding because high concentrations of compounds restrict regulatory approval of medications and cause the medication to be very costly. In addition, the treatment has been well-tolerated as the 149 patients treated in the present study did not present any adverse reaction due to topical application of medications.

## Conclusion

Laser skin resurfacing is a therapeutic modality that can deliver thermal energy to a skin surface to reduce wrinkles, and improve skin tone, texture and pigmentation. The collimation of the laser light on the surface of skin produces micro-channels into the dermis at a homogeneous depth, irrespective the irregularity of the wrinkles. AFXLs can facilitate the bioavailability of molecules through the resultant micro-channels. By interrupting the integrity of the SC, lasers reduce the diffusional path length (membrane thickness) and the superficially applied drug fills the laser channels and penetrates into the dermis.

As the skin is a structure that is subjected to alterations under the scale of micrometers, the results of laser skin resurfacing (LSR) may seem less expressive than surgical procedures aiming at skin rejuvenation. However, this study reinforces the potential of using LADD as an ancillary procedure to enhance facial treatment results. The statistical analysis demonstrates an improvement in periorbital wrinkles in both treatment groups, but the addition of a cosmeceutical containing GFs provided significantly better results. This finding indicates that intradermal direct delivery of GFs, by-passing the epidermis, may protect the GFs from enzymatic action and permit a direct bioactive effect. LADD with vitamin C and GFs has proven to be safe and effective, and there were no unexpected local tissue reactions or adverse systemic reactions to either of the LADD substances being investigated.

## Data Availability

Raw data, additional tables and graphics not included in this version are available for consultation.
